# A Useful Combination of Quantitative Ultrashort Echo Time MR Imaging and a Probing Device for Biomechanical Evaluation of Articular Cartilage

**DOI:** 10.3390/bios11020052

**Published:** 2021-02-17

**Authors:** Takehito Hananouchi, Yanjun Chen, Saeed Jerban, Masaru Teramoto, Yajun Ma, Erik W. Dorthe, Eric Y. Chang, Jiang Du, Darryl D. D’Lima

**Affiliations:** 1 Medical Engineering Laboratory, Department of Mechanical Engineering, Faculty of Engineering, Osaka Sangyo University, Daito Osaka 574-8530, Japan; 2 Shiley Center for Orthopaedic Research and Education at Scripps Clinic, La Jolla, CA 92037, USA; dorthe@scripps.edu (E.W.D.); ddlima@scripps.edu (D.D.D.); 3 Department of Radiology, University of California San Diego, San Diego, CA 92093, USA; justmedic@smu.edu.cn (Y.C.); sjerban@health.ucsd.edu (S.J.); yam013@ucsd.edu (Y.M.); e8chang@ucsd.edu (E.Y.C.); jiangdu@ucsd.edu (J.D.); 4 Division of Physical Medicine & Rehabilitation, University of Utah, Salt Lake City, UT 84108, USA; Masaru.Teramoto@hsc.utah.edu; 5 Research Service, VA San Diego Healthcare System, San Diego, CA 92161, USA

**Keywords:** probing device, mechanical property, articular cartilage, quantitative magnetic resonance imaging

## Abstract

In this study, we combined quantitative ultrashort echo time (UTE) magnetic resonance (MR) imaging and an investigation by a probing device with tri-axial force sensor to seek correlations with mechanical properties of human patellar cartilage for in situ evaluation of biomechanical properties. Cartilage blocks (15 × 20 × 5 mm^3^) were dissected from the patella of six donors; 5 mm square regions of interest from the cartilage blocks were imaged using UTE-MR imaging sequences (T2* and magnetization transfer ratio (MTR)), and mechanical properties were measured using a micro indentation device. Then, the vertical reaction force on the cartilage surface was measured while push-probing forward 3 mm with the probing device at a 30° tilt to the horizontal plane. The results showed a positive correlation between stiffness/elastic modulus and each predictor variable (UTE-T2* (r = 0.240 and 0.255, respectively, UTE-MTR (r = 0.378 and 0.379, respectively), and probing device force (r = 0.426 and 0.423, respectively). Furthermore, multiple linear regression analysis showed the combination of the three predictors had stronger correlation (adjusted r^2^ = 0.314 (stiffness), 0.323 (elastic), respectively). Our results demonstrate the potential for these non- and less- invasive methods for in situ evaluation of the mechanical properties of cartilage tissue.

## 1. Introduction

In situ mechanical property investigation of articular cartilage tissue is one of the most important evaluations for both clinical and basic researches in the orthopaedic surgery. This is because normally the cartilage becomes softer due to the decrease of proteoglycan contents and/or the increase of water content during its degeneration [1,2]. If surgical intervention such as micro-fracture [3] or cartilage replacement [4,5,6] to degenerated or damaged cartilage is necessary, there are the following two potential approaches to know the mechanical property of articular cartridge. In pre-operative and post-operative phases, quantitative magnetic resonance (MR) images is one approach (noninvasive approach) [7,8]. If the quantitative MR images can detect not only lesion area, and they can provide mechanical property of the cartilage tissue, we can match the mechanical property of tissue or material for the cartilage replacement with the mechanical property of marginal area around the lesion. The quantitative MR images can also provide the timing decision to initiate partial or full loading longitudinally after surgery. Then, intraoperative assessment with a surgical instrument by touching the cartilage tissue directly is another approach (less-invasive approach) [1,2].

Among the noninvasive approaches, certain quantitative MR sequences, such as transverse relaxation time (T2 or T2*), and spinlock relaxation time (T1ρ) have been reported for qualitative assessment of the articular cartilage tissue [9,10,11]. Also, relatively new quantitative MR images such as ultrashort echo time (UTE) magnetization transfer (UTE-MT) and UTE-T2* mapping have been reported for diagnosis of early cartilage degeneration [12,13,14,15,16]. However, only a few studies reported on the correlation between quantitative MR images and classical mechanical properties of the articular cartilage [7,8]. 

In the less-invasive approaches, several researchers tried to develop or invent devices to measure mechanical property of the articular cartilage [1,2,8,17,18,19,20]. Although ultrasound and mechanical-based indentation devices [1,2,8,17,18] can measure some mechanical properties of articular cartilage, the device has to be oriented such that the flat device tip surface is parallel to the articular cartilage surface, which is similar to conventional methods of a compression test. The remaining electromechanical indentation device [19,20] which has a round shape at the tip of the device, can be also challenging to control tip contact with the cartilage surface during arthroscopy because relatively larger tip size can obscure the measurements.

**Figure 1:**
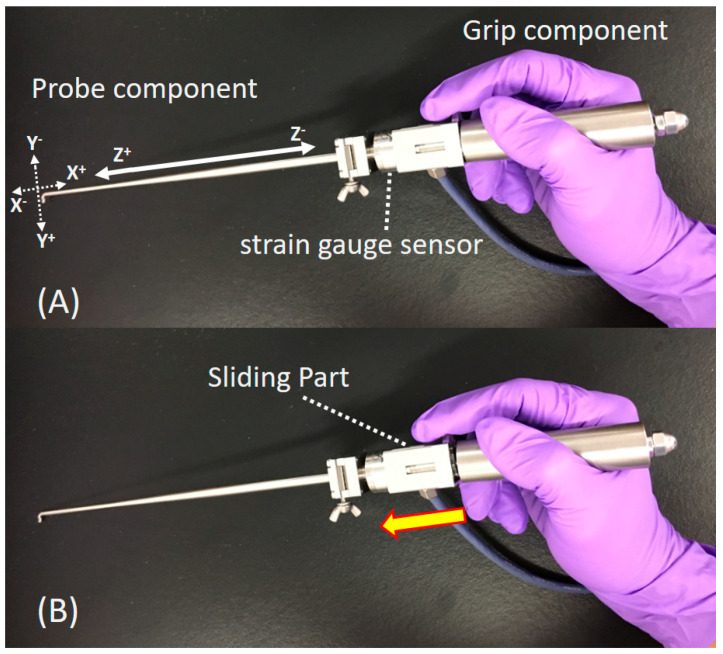
The probing device. The device consists of a probe component with a half-length size and a grip component. A strain gauge sensor is embedded at the top of the grip component, which connects with the probe component (**A**). The sensor on the probe measures force in three axes, indicated by one solid and two dotted arrows, at the tip of the probe. The solid arrow is along the probe axis, and the two remaining dotted arrows are perpendicular to the probe axis. A sliding part (which moves forward in the direction of the yellow arrow) at the grip component has been added to control the distance while pulling and pushing of the probing in the Z direction (**B**).

Recently, one of the authors has developed a probing device with a tri-axial force sensor (Probing Sensor, Takumi Precise Metal Work Manufacturing Ltd., Yao, Japan) [21] to measure the resistance of the soft tissues quantitatively in the joint. The probing device consists of a probe component with half-length size and a grip component (Figure 1). A strain gauge sensor is embedded at the top of the grip component, which connects with the probe component. In one recent paper, the condition of the acetabular labrum (intact, torn, and repaired) of could be differentiated using a prototype of this probing device [22]. A sliding feature at the grip component of the probing device (Figure 1) has been added to control the distance while pulling or pushing the probe [21]. Also, a preliminary study has shown that a vertical reaction force from the probe axis (induced by the probing device) to the surface of cartilage mimic samples (a polyvinyl alcohol hydrogel) while push-probing correlated with mechanical properties by a micro indentation test [21]. However, there is no report on whether the measured values by the probing device correlate with the mechanical property of human articular cartilage.

Therefore, the current study aimed to investigate the correlations between the quantitative MR images (UTE-MT and UTE-T2* mapping) and the force values measured by the probing device, and the mechanical property by the micro indentation test to establish an in situ evaluation of the biomechanical properties of cartilage tissue. We also investigated whether combination by these approaches can detect the mechanical property of the cartilage tissue more precisely to establish an in situ evaluation of the biomechanical properties of the articular cartilage tissue.

## 2. Methods

For this study, six patellae from freshly frozen cadavers (three females, 31–81 years old) were obtained from a non-profit whole-body donation company (United Tissue109 Network, Phoenix, AZ, USA). We took the following two quantitative MR images (MRI), UTE-MT and UTE-T2* mapping on a 3T MRI scanner (MR750, GE Healthcare Technologies, Waukesha, WI, USA) after thawing. Then, we performed a conventional compression test by a customized micro indentation device [21], along with measurement of the reaction force on the cartilage surface by the probing device.

**Figure 2:**
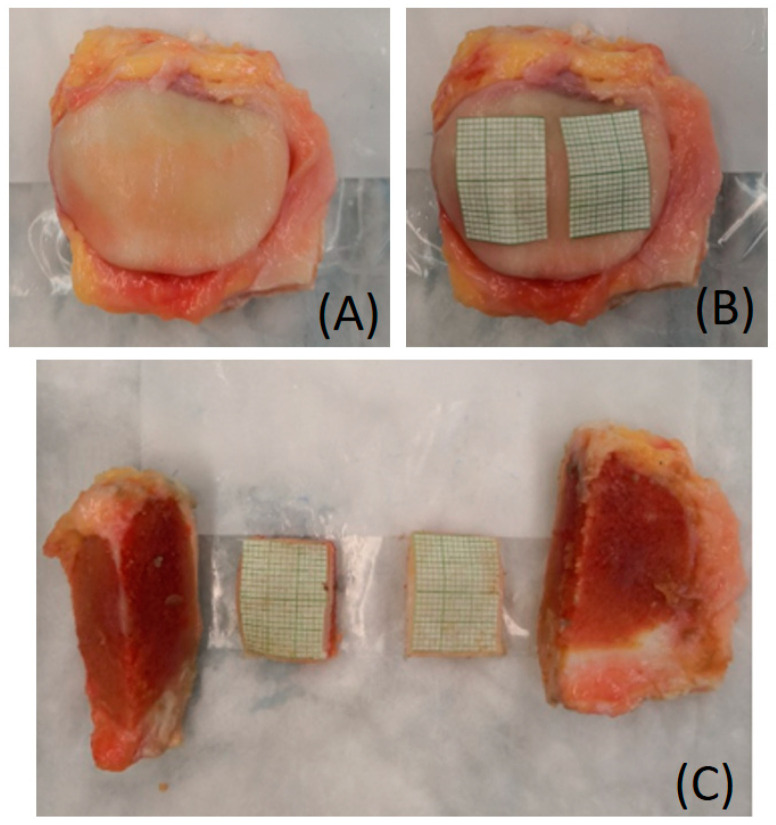
Steps for sample preparation of the patella cartilage; Patella was thawed from a freshly frozen condition (**A**). Two sheets with a grid of 15 mm × 20 mm were placed on the surface of the patella to create a template (**B**). Two 15 mm × 20 mm specimens were prepared from each patella (**C**).

For sample preparation, two cartilage and bone blocks, each nominally 15 × 20 × 5 mm^3^ in dimension, were harvested from medial and lateral parts of each patella. A 15 × 20 mm^2^ paper sheet was placed on medial and lateral sides on each patella, and used as a template to mark the area which was cut on a low-speed precision saw (ISOMET 1000, Buehler, Lake Bluff, IL, USA), (Figure 2). The surface of 15 × 20 mm^2^ per block was partitioned into 5 mm squares to yield 24 segments per patella surface (144 5 mm square segments in all). Specimens were soaked in phosphate-buffered saline for 2 h to ensure adequate hydration throughout the sample preparation. Cartilage blocks were stored into a 30 mL syringe (four blocks per syringe) filled with perfluoropolyether (Fomblin, Ausimont, NJ, USA) to minimize dehydration and susceptibility artifacts during MR imaging.

The MRI scans were performed on a 3T MRI scanner (MR750, GE Healthcare Technologies) using an in-house 1-inch birdcage coil. Two sets of 3D (Three-dimensional) UTE cones MRI sequences were performed. First, a 3D UTE Cones sequence with magnetic transfer (MT) preparation of 800° pulse power level at 2 kHz frequency offset with FA (flip angle) = 7° was performed for MT ratio (MTR) measurement. Second, a 3D UTE Cones T2* sequence was performed with following parameters; TR (repetition time) = 100 ms; FA = 10°; fat saturation; multi-echoes with TEs of 0.032, 5.8, 11.6, 17.4, 23.2, and 29.0 ms, to measure UTE-T2* relaxation time. Other imaging parameters included: field of view (FOV) = 50 mm × 50 mm, matrix = 160 × 160, slice thickness = 0.5 mm, number of slices = 60. Features of the 3D UTE Cones sequence have been described in the previous study [12]. 

**Figure 3:**
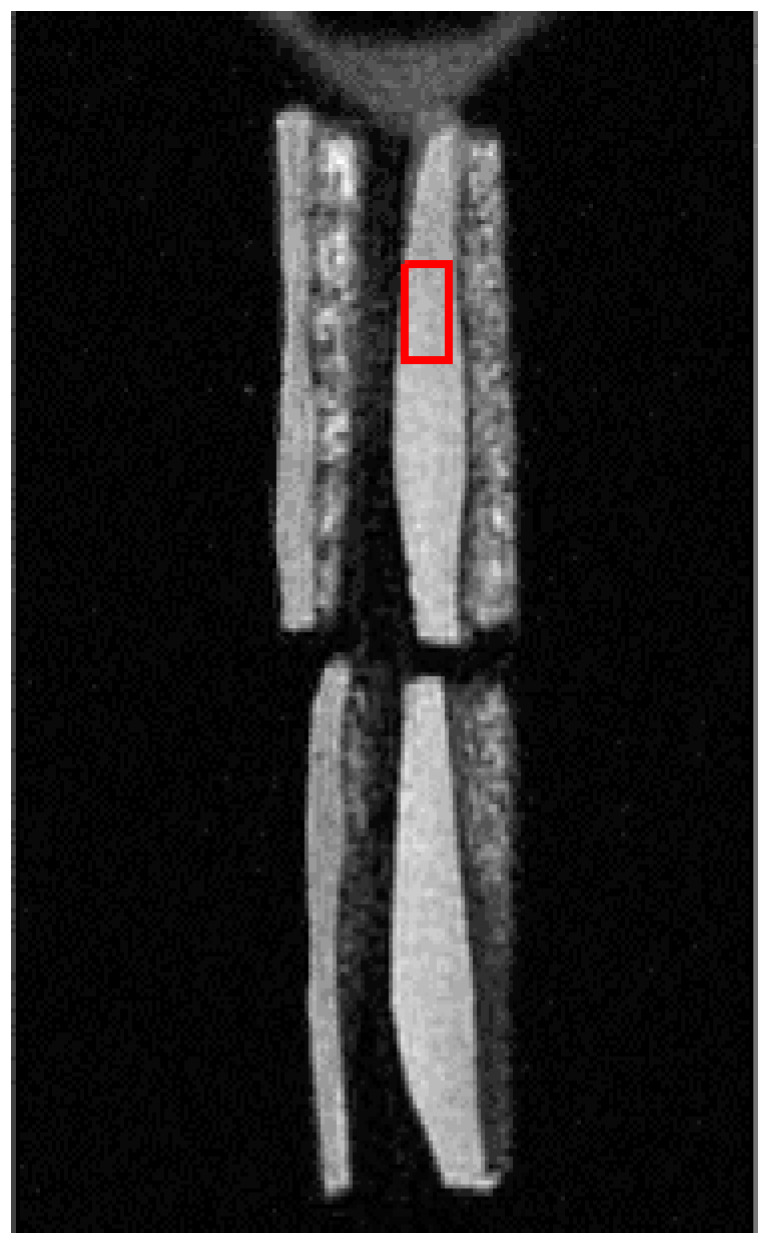
A representative UTE MR image of four patellar cartilage sections to indicate one Region of Interest (ROI). The red rectangle indicates one sample of the ROIs to acquire values of the UTE MR images.

All MR images were acquired in the sagittal planes. Single-component exponential fitting models were used to measure UTE-T2* relaxation times, as described in an earlier study [12]. The acquired UTE-MT dataset was used for MTR value. The above two kinds of MRI values at each 5 mm segment were applied as the MRI values of each 5 mm segment (Figure 3). All MRI calculations were performed using in-house developed scripts in MATLAB (version 2017, The Mathworks Inc., Natick, MA, USA). Also, the cartilage thickness of each 5 mm segment was measured; its value was used for elastic modulus measurement in the following analysis of the microindentation test. The surface angle of each 5 mm segment to the horizontal plane was also measured. Samples of 5 mm segments with surface angles >15° were excluded from the microindentation test. 

**Figure 4:**
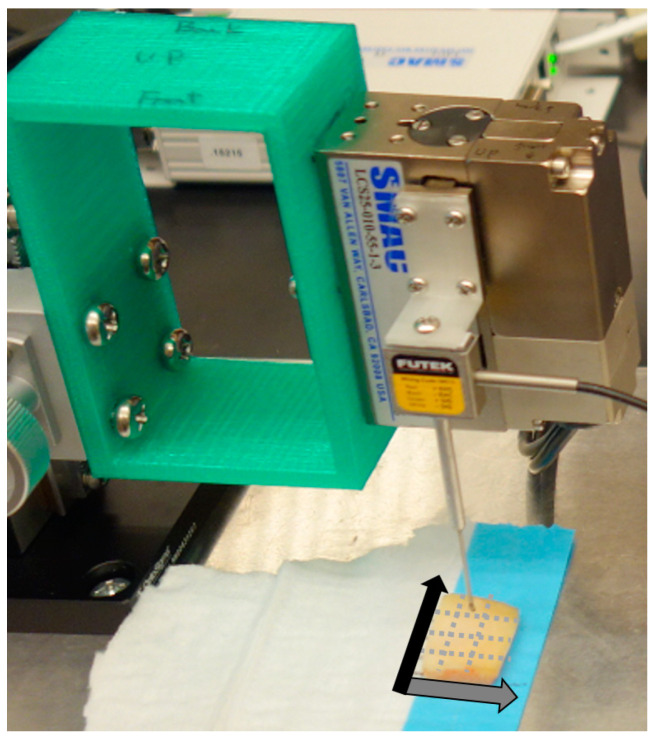
The custom micro indentation device. On each osteochondral sample 12 (3 (in the direction of the gray arrow) × 4 (in the direction of black arrow)) 5 mm square segments were spatially partitioned (dotted gray lines). Stiffness and elastic modulus of each 5 mm square were measured.

The stiffness of the articular cartilage was measured by in-house developed microindentation device with a spherical 1 mm diameter tip, which was explained in the published paper [21] (Figure 4). Each osteochondral block was placed on the baseplate of the indentation device. The midpoint of each 5 mm square segment was aligned with the indenter tip. This measurement method was also followed in the previous paper [21]. The linear portion of the indentation force-displacement curve was used to calculate the stiffness and the elastic modulus, as reported by Hayes et al. [23]. This measurement was done to all 5 mm square segments in a blind fashion; 89 measurements were included for analysis (segments with surface angles >15° were subsequently excluded).

**Figure 5:**
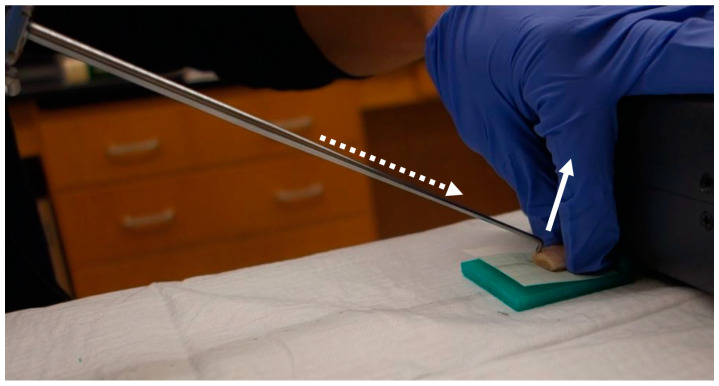
The sensor on the probe measured a reaction force (solid white arrow indicates its direction) on the cartilage surface while manually sliding the probe with 3 mm forward (white dotted arrow indicates its direction).

The reaction force by the probing device to the cartilage surface was measured while push-probing to simulate intraoperative measurement. The grip component has an additional feature that allows for manual sliding with the operator’s index finger (Figure 1). Three components of reaction force are measured by the probing device. The first is along the long axis of the probe. The second is perpendicular to the probe axis along the direction of the hook of the probe. The third is in the transversal direction. The measurement of the forces was conducted using the general method, which was explained in the previous one paper [21]. The force perpendicular to the probe axis along the other direction of the probe’s hook was identified as a partial reaction force to the cartilage surface while pushing the probe at a 30° tilt to the horizontal line (Figure 5). The sliding distance of the grip component of the probing device was set to 3 mm during the push-probing. The probing forces were recorded at a sampling rate of 10 Hz. We measured the reaction force twice and used the average value of the maximum forces recorded during the push-probing, in order to investigate the correlation with cartilage mechanical properties by the micro indentation test. Each specimen block was kept moist with saline solution during the mechanical investigations.

Descriptive statistics were calculated for stiffness and elastic modulus (= outcome variables), along with UTE-T2*, UTE-MTR, and probing device force (= predictor variables). Associations of stiffness and elastic modulus to each predictor were examined using Pearson correlation coefficient (r), as well as scatterplots. The effect size of r was determined as follows: 0.10 = small effect, 0.30 = medium effect, and 0.50 = large effect [24]. Further, multiple linear regression (MLR) analysis was performed to examine if UTE-T2*, UTE-MTR, and probing device force could predict stiffness and elastic modulus. Model fit was assessed using adjusted R2 and root mean square error (RMSE). Semipartial correlation coefficient (sr) and squared semipartial correlation coefficient (sr2) were also calculated to quantify the contribution of each predictor to the regression models.

## 3. Results

A total of 93 specimen blocks were analyzed in this study. Descriptive statistics for stiffness elastic modulus, UTE-T2*, UTE-MTR, and probing device force are summarized in Table 1.

**Figure 6:**
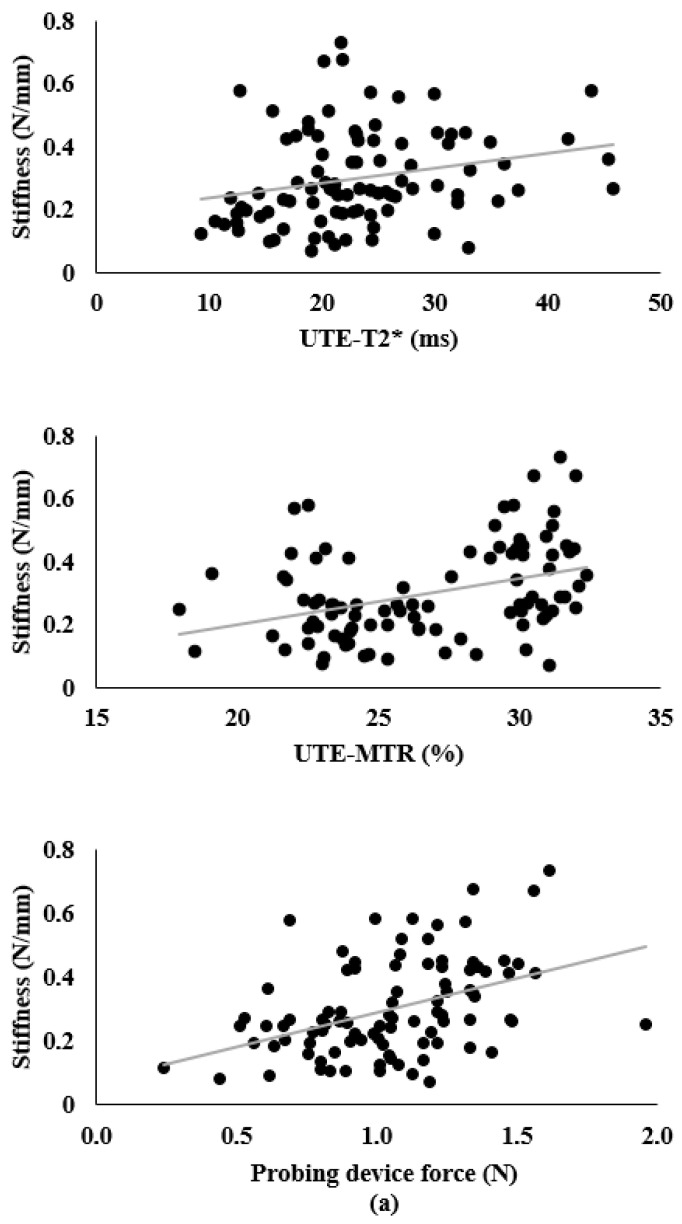

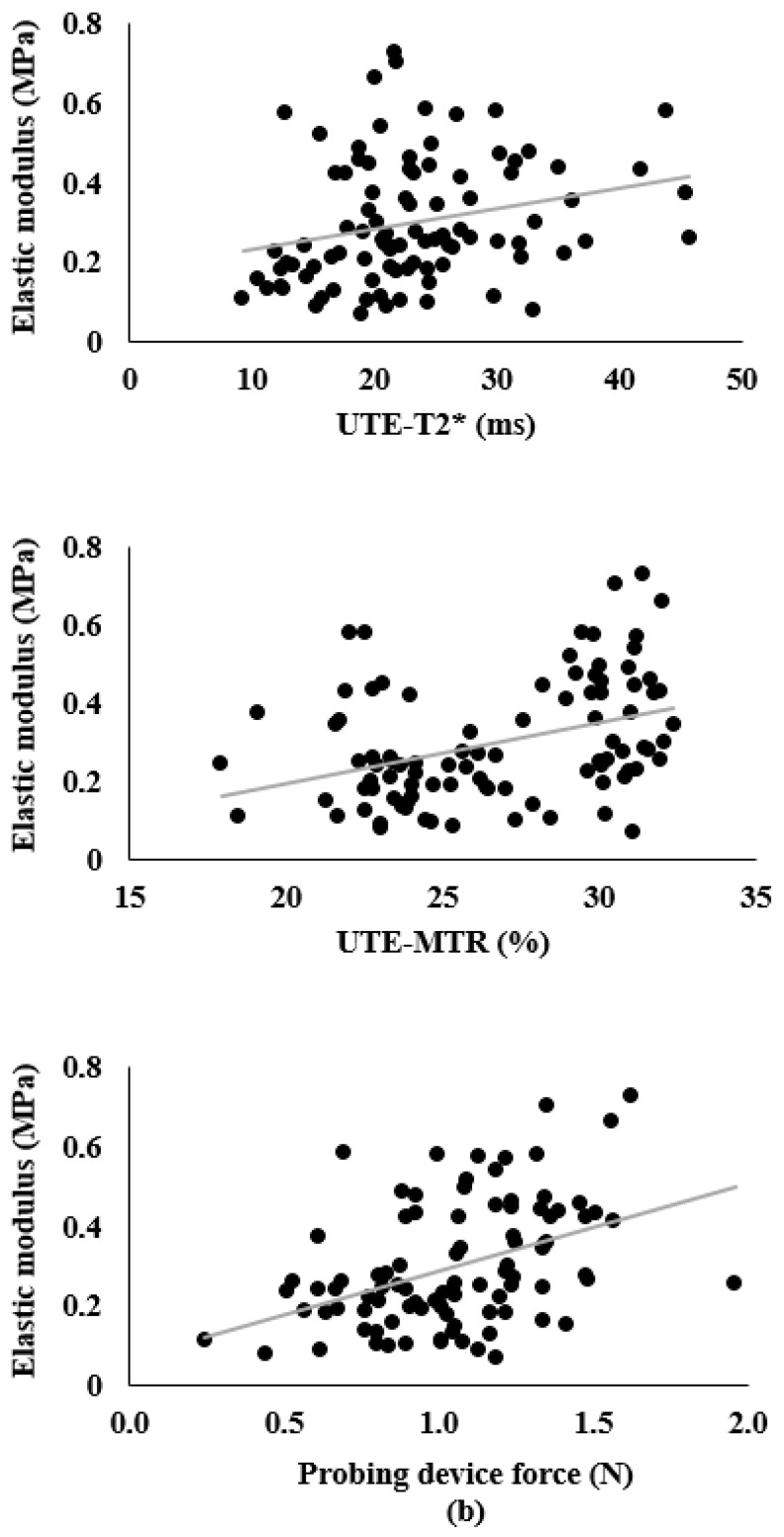
Scatterplots of: (**a**) stiffness by ultrashort echo time transverse relaxation time (UTE-T2*), ultrashort echo time magnetization transfer ratio (UTE-MTR), and probing device force, and (**b**) elastic modulus by UTE-T2* UTE-MTR, and probing device force.

Associations among the variables are shown in Table 2 and in Figure 6a,b. There was a positive correlation between stiffness/elastic modulus and each predictor variable. Specifically, positive, small-sized correlations were observed between stiffness/elastic modulus and UTE-T2* (r = 0.240 and 0.255, respectively). Additionally, positive, medium-sized correlations were observed between stiffness/elastic modulus and UTE-MTR (r = 0.378 and 0.379, respectively), and probing device force (r = 0.426 and 0.423, respectively). In addition, the average differences of the two measurements by the probing force on each specimen block was 0.1 N.

**Figure 7:**
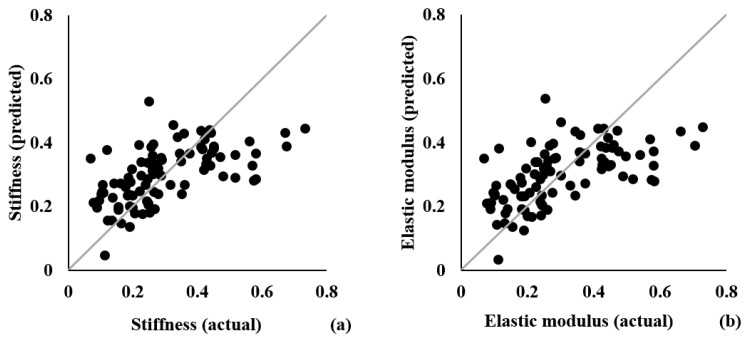
Scatterplots of actual vs. predicted values on stiffness (**a**) and elastic modulus (**b**). Gray line = line of identity.

The results of the MLR analysis are summarized in Table 3. The two models were able to explain over 30% of the variability in stiffness (adjusted r^2^ = 0.314, RMSE = 0.124) and elastic modulus (adjusted r^2^ = 0.323, RMSE = 0.128). Further, all three predictors, UTE-T2*, UTE-MTR, and probing device force, were significant to predicting stiffness (*p* = 0.001 for all three predictors) and elastic modulus (*p* < 0.001 or = 0.001 for all three predictors). According to sr and sr^2^, the contribution of each predictor to the model for stiffness was similar, accounting for about 8–10% of the variability in the model. Similar contributions were also found in the model for predicting elastic modulus, accounting for about 9–11% of the variability in the model. Actual vs. predicted values on stiffness and elastic modulus are depicted in Figure 7.

## 4. Discussion

One of the most important findings in the current study are the correlations between the UTE-MTR value and UTE T2* relaxation time and the mechanical property of the articular cartilage. These UTE values may therefore be able to predict the in situ mechanical properties of the articular cartilage tissue in clinical situations before surgery. The efficacy of UTE-MTR and/or UTE-T2* in predicting mechanical properties has been reported in ex vivo cortical bones (femur and tibia) [25,26], distal femoral cartilage [12,13,14], Achilles tendon [27], rotator cuff tendon [28], and in vivo tibial tendons [29]. UTE-MTR and UTE T2* values also strongly correlated with histological grades (Mankin scores) of cartilage degeneration (UTE-MTR; r = −0.678, UTE-T2*; R = −0.501) [12]. Our findings for mechanical properties of articular cartilage further support the application of UTE-MTR and the UTE-T2* for the diagnosis of early cartilage degeneration.

Articular cartilage is a dense avascular connective tissue, consisting of relatively few cells embedded in a highly charged and hydrated extracellular matrix (ECM), composed of collagen, PGs, and water [30]. The degeneration of cartilage occurs due to the loss of PG and collagen in the cartilage matrix. Since the MTR and T2* values are proportionally correlated with the amount of collagen and water content, the UTE-MTR and UTE-T2* values are highly sensitive in detecting cartilage degeneration. Because macromolecular and water content are major factors contributing to tissue biomechanics, UTE sequences may also correlate with load bearing capacity of articular cartilage. UTE-MTR was found to be more sensitive than the UTE-T2* in detecting early degeneration of the articular cartilage [12].

According to some previous studies about the mechanical properties and the histological assessments of the articular cartilage, the more degenerated cartilage tissue had the low compressive dynamic stiffness [2,31,32]. In one paper using the most extensive samples of the human patellar cartilage with about 70 samples of 14 patellae [2], the compressive dynamic stiffness of the cartilage by a small mechanical indentation device with semi-spherical indenter (ø = 0.5 mm) was 0.41 ± 0.18 N in healthy samples, 0.40 ± 0.18 N in early degeneration samples, and 0.23 ± 0.16 N in advanced degeneration samples. Therefore, the above results concerning the correlation between the mechanical properties of articular cartilage and the quantitative magnetic resonance imaging in the current study must be explored.

Another significant finding in the present study was the moderately strong relationship between the reaction force documented by the probing device, and the elastic modulus and the stiffness calculated during micro indentation. In the preliminary study with samples of a cartilage mimic (a polyvinyl alcohol hydrogel), there was a stronger relationship between the reaction force by the probing device and the mechanical properties by the micro indentation device (the reaction force by the probing sensor vs. elastic modulus, R = 0.965 and *p* = 0.0044; the reaction force vs. stiffness, R = 0.975 and *p* = 0.0021) [21]. This stronger correlation is likely due to the more homogenous material properties of and the consistent thickness. We postulated that this probing device can provide an in situ mechanical property evaluation of the cartilage tissue under arthroscopy. During arthroscopy, surgeons probe the surface of the articular cartilage to get a subjective impression of the health of the tissue. Since the design of the probing device adheres closely to the shape of the classical surgical probe, the probing device can reproduce conditions of conventional probing and can therefore quantify the more subjective “surgeon’s feeling.” Our results provide rationale for how a quantitative probing device during arthroscopy can be beneficial for measuring specific mechanical properties in joints during arthroscopy. Besides, the measurement by the probing device has been applied to a conventional and known consecutive and quantitative parameter unit (i.e., Newton). Therefore, it can be understood easily by surgeons.

We are still in the initial phase of development of the probing device. Further work is required to establish a robust system of evaluation of mechanical properties under arthroscopy, ideally, only by using the probing device. For surgical application in vivo, it is necessary to control the angle between the cartilage surface and the probing-axis. In our study, the support object was used to set the angle and position of the probing. Our intention was to reproduce intraoperative conditions during manual arthroscopy surgery. The minimal differences in the reaction force between the repeated measurements, indicates it is feasible to control the angle and the position for the probing if there is a support object to keep the surgeon’s hand steady. Furthermore, three-dimensional registration of the angle and the position of the probing device will help to enhance accuracy and repeatability during sequential evaluations. Incorporating sensors that measure the angle and the position three-dimensionally may help address this limitation [33,34,35]. 

The two different non-destructive approaches for in situ mechanical property evaluation of the articular cartilage were tested in the current study. A discussion is now needed on which approach is most beneficial, however, further data and evidence is needed. This is because the selection of the approaches is dependent on various situations during the cartilage replacement The UTE sequences can be very useful for preoperative evaluation and postoperative periodic follow-up. On the other hand, in first and/or second-look operations, the probing device can be useful. Recently, a small arthroscopy system has been developed and commercialized by Nano Arthroscopy Cameras (Arthrex, Fl USA; URL: https://www.arthrex.com/imaging-resection/nano-arthroscopy-cameras). If the size of the probing device can be modified, it may be feasible for potential investigation in outpatient situations with a careful combination of the two modalities.

There are several limitations to the current study. First, this study was performed ex vivo (at room temperature and extracellular pH), and the measurement conditions were different from the in vivo environment. This is a limitation associated with most ex vivo studies before translating to clinical use. A more comprehensive evaluation of cartilage tissue should be investigated in future studies under diverse conditions. Second, the UTE-MTR values are susceptible to many factors, including deviation frequencies, radiofrequency angles, and field intensities. These factors can introduce small discrepancies of MTR values, affecting evaluation of cartilage degeneration. However, the values of the two quantitative MR images were similar to those in our previous study [12]. Third, the authors should have investigated the mechanical property of the articular cartilage in later phase to acquire ‘stress-strain’ relationship as another standard technique [36]. Although the authors followed the method to investigate the mechanical property in the previous paper by one of the authors [21], further studies to investigate several kinds of the mechanical property are needed.

In conclusion, we were able to correlate the mechanical property of the articular cartilage of the patellae by the micro indentation test with the quantitative MR images, i.e., UTE-MTR and UTE T2* and the mechanical investigation by the probing device with the force sensor. The non- and less-invasive evaluation in our study can be beneficial for the in situ mechanical evaluation of the articular cartilage during arthroscopic treatment.

## Figures and Tables

**Table 1:** Descriptive statistics for outcome and predictor variables (*N* = 93).

Variable	Mean (SD)
Stiffness (N/mm)	0.30 (0.15)
Elastic modulus (MPa)	0.30 (0.16)
UTE-T2* (ms)	23.18 (7.70)
UTE-MTR (%)	26.76 (3.80)
Probing device force (N)	1.06 (0.30)

UTE-T2* = Ultrashort echo time transverse relaxation time; UTE-MTR = ultrashort echo time magnetization transfer ratio.

**Table 2:** Pearson correlation coefficients among variables.

Variable	Stiffness	Elastic Modulus	UTE-T2*	UTE-MTR	Probing Device Force
Stiffness	1.000	-	-	-	-
Elastic modulus	0.997	1.000	-	-	-
UTE-T2*	0.240	0.255	1.000	-	-
UTE-MTR	0.378	0.379	−0.174	1.000	-
Probing device force	0.426	0.423	−0.063	0.356	1.000

UTE-T2* = Ultrashort echo time transverse relaxation time; UTE-MTR = ultrashort echo time magnetization transfer ratio.

**Table 3:** Multiple linear regression models on predicting stiffness and elastic modulus.

Outcome Variable	Predictor	B	95% CI	p	sr	sr^2^
Stiffness ^a^	UTE-T2*	0.006	0.003–0.010	0.001	0.311	0.097
	UTE-MTR	0.012	0.005–0.020	0.001	0.289	0.084
	Probing device force	0.168	0.076–0.260	0.001	0.312	0.097
	Intercept	−0.351	−0.567, −0.135	0.002	-	-
Elastic modulus ^b^	UTE-T2*	0.007	0.003–0.010	<0.001	0.326	0.106
	UTE-MTR	0.013	0.005–0.021	0.001	0.294	0.086
	Probing device force	0.173	0.077–0.268	0.001	0.309	0.096
	Intercept	−0.387	−0.610, −0.165	0.001	-	-

UTE-T2* = Ultrashort echo time transverse relaxation time; UTE-MTR = ultrashort echo time magnetization transfer ratio; B = beta coefficient; CI = confidence interval; sr = semipartial correlation coefficient; sr^2^ = squared semipartial correlation coefficient, ^a^F(3, 89) = 15.04, *p* < 0.001, r^2^ = 0.336, adjusted r^2^ = 0.314, root mean square error = 0.124, ^b^F(3, 89) = 15.61, *p* < 0.001, r^2^ = 0.345, adjusted r^2^ = 0.323, root mean square error = 0.128.
